# Consequences of Abdominal Adiposity within the Metabolic Syndrome Paradigm in Black People of African Ancestry

**DOI:** 10.3390/jcm3030897

**Published:** 2014-08-13

**Authors:** Trudy Gaillard

**Affiliations:** Division of Endocrinology, Diabetes and Metabolism, The Ohio State University Wexner Medical Center, 541 McCampbell Hall, 1581 Dodd Drive, Columbus, OH 43210, USA; E-Mail: trudy.gaillard@osumc.edu; Tel.: +1-614-685-3363; Fax: +1-614-685-3329

**Keywords:** obesity, abdominal adiposity, metabolic syndrome

## Abstract

The metabolic syndrome (MetS) is a constellation of risk factors that are associated with increased risks for coronary heart disease and type 2 diabetes. Although the cause is unknown, abdominal adiposity is considered the underpinning of these metabolic alterations. Hence, increased abdominal adiposity contributes to dyslipidemia, hyperglycemia, beta cell dysfunction, insulin resistance, hypertension and inflammation. The role of abdominal adiposity in the causation of metabolic alterations that lead to the clinical expression of the MetS has become a focus of active research. In addition, there are ethnic/racial differences in the manifestation of the MetS. Therefore, the focus of this current review is to: (1) explore the consequences of abdominal obesity within the MetS paradigm; and (2) discuss the impact of ethnicity/race on MetS in Black People of African Ancestry (PAA).

## 1. Introduction

During the past 20 years, there has been a dramatic increase in obesity in the United States. More than one-third of U.S. adults (35.7%) are obese and two-thirds (69%) are overweight [1,2]. In addition, approximately 17% (or 12.5 million) of children and adolescents, aged 2–19 years are obese according to data from the National Health and Nutrition Examination Survey, 2009–2010 (NHANES) [2]. Recent reports show that substantial differences exist in obesity prevalence segregated by race/ethnicity, and these differences vary by sex and age. For example, according to 1999–2010 data from the National Health and Nutrition Examination Survey, which used body mass index (BMI) of 30 or greater (calculated as weight in kilograms divided by height in meters squared), the overall age-adjusted prevalence of obesity among adults was 35.7%. Among men, the age-adjusted obesity prevalence was 35.5% (36.2% for white men and 38.8% for African American men). For women, the age-adjusted prevalence was 35.8% (32.2% for white women and 58.8% for African American women). It has been noted that the overall prevalence of obesity did not differ significantly between men and women during this time frame [1,2]. 

Studies examining trends in obesity and overweight in Sub-Saharan Africa (SSA) are rare. Existing studies compare mostly prevalence studies on individual countries and in sections of the populations. Such studies are often not based on representative samples and have varying methodological problems that make these findings difficult to generalize to the entire SSA population. In the context of these limitations, it is difficult to comment on the distribution of obesity in SSA populations. Nonetheless, some studies on the prevalence of overweight and obesity in SSA populations suggest similar rates to those reported in the industrialized world, specifically for women [3,4,5,6,7,8,9]. Recent studies by Ziraba *et al.* [6] found that the prevalence of overweight and obesity grew by almost one-third between 1992 and 2005 in a sample of women from SSA countries. Fifty-six percent of South African women and 29% of men were overweight or obese according to a 2002 study [8].

Recently, the World Health Organization (WHO) has estimated that by 2015, 2.3 billion adults will be overweight and 700 million adults will be obese [10]. Sub-Saharan Africa is not immune to the obesity epidemic, despite the continued burden of under nutrition in many SSA countries. Delisle *et al.* [9] in a cross-sectional study on nutrition transition and cardiometabolic risk factors in both overweight (35.5%) and underweight (11.3%) apparently healthy adults in Benin, West Africa suggested that chronic energy deficiency in either circumstance maybe related to accelerated cardio-metabolic risk factors in SSA countries. Increases in the rates of overweight and obesity are being identified in SSA, especially among women and people dwelling in urban populations [3,4,5,6,7,8,9].

## 2. Criteria of Metabolic Syndrome Using the National Cholesterol Education Program—Adult Treatment Panel III

Obesity constitutes a putative risk for cardiovascular disease (CVD) and type 2 diabetes as defined by the Metabolic Syndrome (MetS). The MetS is a clustering of metabolic traits that increase an individual’s risks for future development of type 2 diabetes and cardiovascular disease [11,12,13,14]. It includes five components that are associated with increased risk of these diseases. These include abdominal obesity (measured as waist circumference or body mass index (BMI)); hypertension (raised systolic or diastolic blood pressure); dyslipidemia (low high-density lipoprotein cholesterol (HDL-C) and high triglyceride levels) and dysglycemia (high fasting glucose). Various diagnostic criteria have been proposed for the MetS by several organizations including the World Health Organization (WHO) [11], the European Group Insulin Resistance (EGIR) [12], the National Cholesterol Education Program Adult Treatment Panel III (NCEP-ATP-III) [13], the International Diabetes Federation (IDF) [14] (Table 1). Any three of the five components signify MetS and suggest further follow-up by the primary health care providers. The NCEP-ATP III criteria for MetS is more commonly used in the U.S. Using NCEP-ATP III criteria and the 2000 USA census data, there were approximately 47 million U.S. adultswith MetS [15,16]. When comparing NHANES I and II, the prevalence of MetS increased from 23%, to 26.7% respectively [15,16]. The NHANES III also revealed that there are racial/ethnic differences in the prevalence and incidence of MetS in USA. According to NHANES III, the prevalence of MetS was 13.9% for African American men and 20.9% for African American women during 1988–1994 [15,16]. The corresponding rates of MetS were 25% in white men and 23% white women for the same time period. The reasons for the racial/ethnic differences in CVD risk factors and lower MetS in African Americans remain unknown. Based on the higher CVD outcomes in African Americans, we surmised that there are also racial/ethnic differences on the impact of the five components of MetS for future CVD and type 2 diabetes. Most importantly, because obesity is more common in African Americans, particularly African American women and black women across the African Diaspora, it is important to examine each of the components in combination with obesity.

**Table 1:** Definitions of metabolic syndrome by various organizations.

	National Cholesterol Education Program. Report of the Adult Treatment Panel III (R)	WHO Metabolic Syndrome Definition 1999	European Insulin Resistance Group	International Diabetes Federation
**Special Features**	**None**	**Insulin Resistance, Type 2 diabetes IFG, IGT**	**Insulin Resistance Top 25% of fasting insulin**	**Abdominal obesity Waist Circumference** Men > 94 cm Women > 80 cm
	Any 3 of the following:	Plus any 2 of the following:	Plus any 2 of the following:	Plus any 2 of the following:
**Abdominal obesity**	Waist Circumference Men > 102 cm Women > 88 cm	Waist Circumference Men > 102 cm Women > 88 cm or BMI > 30 kg/m^2^ or WHR Men > 0.9% Women > 0.85%	Waist Circumference Men > 94 cm Women > 80 cm	**Requirement** or BMI > 30 kg/m^2^ or WHR Men > 0.9% Women > 0.85%
**Blood pressure**	>130/80 mmHg Medication Usage	>140/90 mmHg Medication Usage	>140/90 mmHg Medication Usage	>140/90 mmHg Medication Usage
**Glucose**	>100 mg/dL	**Required**	>110 mg/dL	Insulin Resistance, Type 2 diabetes, IFG, IGT
**Lipids** **a** **b**	HDL Men < 40 mg/dL Women < 50 mg/dL Triglycerides >150 mg/dL	HDL Men < 40 mg/dL Women < 50 mg/dL Triglycerides >150 mg/dL	HDL <40 mg/dL Triglycerides >160 mg/dL	HDL Men < 40 mg/dL Women < 50 mg/dL Triglycerides >150 mg/dL
**Other**	None	Urinary albumin >20 mg/min: Alb/Cr > 30 mg/g	Ethnic differences in WC measures	Urinary albumin >20 mg/min: Alb/Cr > 30 mg/g
**Reference Number**	[13]	[11]	[12]	[14]

IFG = impaired fasting glucose; IGT = impaired glucose tolerance; BMI = body mass index; Alb/Cr = albumin/creatinine ratio.

Recent studies have shown that African Americans and black people of African Ancestry (PAA) are more insulin resistant, the proposed pivotal lesion of the MetS, and more obese when compared to their white counterparts, regardless of geographical location [17,18,19,20,21,22,23,24,25]. However, the relationships among insulin resistance and the various metabolic components of MetS are complex and confounded by genetic, environmental and ethnic factors. However, the relationships among obesity and the major components of MetS have not been adequately studied in PAA. Therefore, there is great need to explore the relationships of obesity and components of the MetS in PAA.

### 2.1. Metabolic Syndrome in Black People of African Ancestry

The prevalent rates of MetS vary among PAA. For example, the prevalence of MetS was 13.9% for African American men and 20.9% for African American women during 1988–1994 in NHANES [15,16]. Variable results have been demonstrated in blacks in other diverse geographic regions. In the Jackson Heart Study, Taylor *et al.* [26] found the overall prevalence of MetS in African Americans was 39.4% (43.3% in women and 32.7% in men). Similarly, Gaillard *et al.* [27], reported the prevalence of MetS in African American women with family histories of type 2 diabetes to be 35.5%.

Several authors have also studied the prevalence of MetS in various SSA populations. Oladapo *et al.* [28] found the prevalence of MetS to be only 2.1% in men and 2.7% in women of rural Yoruba south-western Nigerian population. Adediran *et al.* [29] studied the prevalence of MetS in rural and urban Abuja, Nigeria. They found the prevalence of MetS to be 3.7% in rural Nigeria *vs.* 13.7% in the city of Abuja. Similar findings, have been reported in rural (0%–1.9%) *vs.* urban (0.2%–7.3%) Cameroonians [8] and in rural (4.1%), semirural (6.4%) and urban (11%) Africans from Benin [30]. Clearly, there are rural and urban gradients in the prevalent rates of Mets in PAA. Other studies in SSA found higher prevalence rates of MetS. For example, Katchunga *et al.* [31] found the MetS prevalence to be 43.6% among Congolense with at least one major cardiovascular risk factor. Note however, that in this study, the subjects were recruited based on having one prior CVD factor. In urban dwelling Tanzanian subjects, MetS was observed in 38% of their population using IDF definition [32]. In another study of Botswana hospital health care workers, 34% were found to have MetS using IDF criteria [33], with a high prevalence of obesity (28.7%) and overweight (27.3%) in their study. Jennings *et al.* [34] in a population of overweight and obese South African women found the prevalence of MetS to range from 5.3% to 21.9% using either ATP III or IDF definition. Similarly, Kelliny *et al.* [35] found the prevalence of MetS to vary between the three definitions used in a population of Africans in the Seychelles Islands of East Africa with 24%, 25%, 25.1% in men and 32.2, 24.6 and 35.4 using NCEP-ATP III, WHO and IDF definitions, respectively. Finally, Tillin *et al.* [36] in population-based cross-sectional study done in the United Kingdom, compared the ethnic and sex differences between the WHO and NCEP definition of MetS. In particular, both definitions of MetS predict CVD risk in European, but not equally in South Asian or African-Caribbean populations (Table 2).

**Table 2:** Summary of metabolic syndrome in various populations of people of African ancestry.

Author	Definition of Metabolic Syndrome Used	Prevalence of Metabolic Syndrome	Male	Female	Country	Reference
Adediran	NCEP-ATP III	13.7 rural 3.7 urban	-	-	Nigeria, West Africa	[29]
		3.7 urban			West Africa	
Fezeu	IDF	Rural	0.0	0.3	Cameroon, West Africa	[8]
		Urban	1.2	1.5		
	NCEP-ATP III	Rural	0.0	0.0		
		Urban	0.5	0.2		
	WHO	Rural	1.9	1.8		
		Urban	7.3	5.9		
Ford	NCEP-ATP III	21.6	16.4	25.7	National Sample, USA	[15]
Gaillard	NCEP-ATP III	35	-	35.5	Columbus, OH, USA	[27]
Garrido	IDF	34	15.9	24.5	Botswana, Southern Africa	[33]
Gyakobo	IDF	35.9	15.7	55.8	Ghana, West Africa	[30]
	NCEP-ATP III	15.0	5.9	24.0		
Jennings	IDF	21.9		12.9	South Africa	[34]
	NCEP-ATP III	5.3	-	10.4		
Katchunga	IDF	49.0	31.2	59.0	Demographic Republic of Congo, Central Africa	[31]
	NCEP-ATP III	43.6	30.9	52.2		
Kelliny	IDF	30.3	25.1	35.4	Seychelles Island, West Africa	[35]
	NCEP-ATP III	28.1	34.0	32.2		
	WHO	24.5	25.0	24.6		
Meis	NCEP-ATP III	30.0	40.0	29.0	Columbus, OH, USA	[19]
Motala	JIS	26.5	11.6	30.3	South Africa	[23]
Njelekela	WHO	38.8	36.2	53.0	Tanzania, West Africa	[32]
Ntandou	IDF	Rural 4.1	0	8.2	Benin, West African	[30]
		Semi-rural 6.4	4.7	8.2		
		Urban 11	5.0	17.0		
Oladapo	NCEP-ATP III	3	2.1%	2.7%	Nigeria, West Africa	[28]
Taylor	NCEP-ATP III	39	32.7	43.3	Jackson, MS, USA	[26]
Tillin	NCEP-ATP III	32	26.7	26.4	United Kingdom	[36]
	WHO	18	15.5	23.4		

IDF—International Diabetes Federation; JIS—Joint Interim Statement; NCEP-ATP III—National Cholesterol Education Program—Adult Treatment Panel III; WHO—World Health Organization.

These various studies in blacks confirm that there are ethnic and gender as well as geographical differences in the prevalence of MetS in PAA and its ability to identify individuals at greater risk for CVD when compared to whites residing in similar locations. These observations provide the impetus for redefining the importance of MetS and its various components as predictors of CVD and type 2 diabetes among black people in the African Diaspora.

### 2.2. Role of Insulin Resistance in Metabolic Syndrome in Black PAA

The well-known pivotal lesion underpinning MetS is insulin resistance [11,12,13,14,17,21,25]. The insulin resistance is greater in PAA than whites [17,18,19,20,21,25]. However, the relationships among insulin resistance and CVD risk factors such as hypertension, high density lipoprotein cholesterol (HDL-C) and triglycerides and obesity are very weak in PAA at best, when contrasted with whites. The paradox of more favorable lipid profile and conversely the higher rates of unfavorable blood pressure in PAA calls into question the current criteria or cut-off points for MetS in PAA. In aggregate, it can be argued that each of the components of the MetS carry different CVD risks factors in blacks PAA in general [22]. The role of insulin resistance and the various components of the MetS will be discussed in further detail in the following sections.

### 2.3. Paradox of Insulin Resistance, Body Composition and Metabolic Syndrome in Black PAA

Obesity and type 2 diabetes have become epidemic in blacks in the western world in U.S., UK and West Indies. Both diseases are undoubtedly increasing in SSA developing countries. Both diseases contribute significantly to CVD in blacks and whites [17,18,19,20,21,31,32,33,34,35,36]. In particular, PAA, especially women, have greater rates of CVD morbidity and mortality than white women probably due to higher rates of obesity, type 2 diabetes, hypertension, strokes and congestive heart failure and physical inactivity [22,25,36]. However, and surprisingly, the relatively higher HDL-C levels in the face of lower triglycerides levels even in obese, insulin resistant black subjects do not appear to be cardio-protective of coronary artery disease (CAD) in blacks when compared with whites.

A major determinant of insulin resistance is abdominal visceral adiposity. It is well established that visceral adiposity of the abdomen is a powerful predictor or determinant of not only insulin resistance, but metabolic syndrome and atherogenic lipids and lipoproteins in several populations. However, in obese women of African ancestry, there is only poor and weak relationships between insulin sensitivity, and lipids and lipoprotein and blood pressure [19,20,21,25,26,27,32,33,34,37,38,39,40]. Furthermore, African Americans with identical BMI have lower visceral adiposity than their white counterparts, even though the former manifests greater insulin resistance. Studies conducted by Albu *et al.* [41], Conway *et al.* [42] and Lovejoy *et al*. [43] found that African Americans with identical body weight and BMI had lower percent of visceral adiposity when compared to their white counterparts. This relationship persisted after adjustments for insulin resistance. Recently, Jennnings *et al.* [34] and Crowther *et al.* [39] have also confirmed lower visceral adiposity in South African black women than South African white women. These studies clearly demonstrated that there are paradoxical and inconsistent relationships between visceral adiposity, insulin sensitivity and body composition as well as, blood pressure in PAA. In addition, Hoffman *et al*. [44] performed CT scans of the abdomen in healthy African Americans and whites. The authors demonstrated that both African American men and women had lower percent visceral adipose tissue when compared to whites. The subcutaneous adipose tissue was similar among the populations. Given the limited economic resources in SSA, understanding the clinical and metabolic correlates of obesity in black PAA, especially women who are either at risk or more prone to CVD and type 2 diabetes will be important in determining strategies for primary prevention of CVD and type 2 diabetes in PAA.

### 2.4. Metabolic Syndrome and Lipids and Lipoprotein’s in Black PAA

Several large population studies consistently show that serum HDL-C levels are very important and independent predictors of CAD in non-black populations [16,20,21]. Black people of African ancestry often have higher levels of HDL-C than their white counterparts. Thus, theoretically, and based on these previous studies, the extraordinarily lower levels of serum triglycerides (in the face of elevated HDL-C) in obese, insulin resistant, black PAA when compared to whites should result in lower CAD and CVD outcomes [19,20,21,26,36,37,38]. However, this is not the case in nondiabetic PAA. Thus, the relatively favorable lipid and lipoprotein levels in PAA can partly explain the lower prevalent rates of MetS observed in PAA.

We have previously shown that African Americans manifest greater insulin resistance and hyperinsulinemia when compared to white Americans [17,18,19,37]. Most importantly, African Americans with greater insulin resistance also paradoxically have relatively higher HDL-C and lower triglycerides levels when compared to their white counterparts [15,16,19,20,21,26,27]. Recently, Haffner *et al.* [20] have shown that in addition to relatively lower triglycerides and higher HDL-C levels, African Americans have also larger LDL-particles sizes when compared to whites in the Insulin Resistance Atherosclerosis Study (IRAS). Despite, these favorable anti-atherogenic lipid and lipoprotein profiles, African Americans continue to suffer enormously and disproportionately from higher CVD morbidity and mortality than white Americans. Thus clearly, the favorable lipid and lipoprotein profile does not appear to protect African Americans against excessive CVD mortality and morbidity [22,25]. The reasons are unclear. In another study examining the cardiometabolic health of African American, West African and Central African men found similar HDL-C and triglycerides profile among the groups. Surprisingly, the African Immigrants had worse cardiometabolic risk than the African American men in their study [45]. Some of the reasons for the more favorable lipid profile in black PAA have been related to the higher lipoprotein lipase activity found in black PAA [46,47]. We believe that the thresholds for CVD metabolic risk factors in African Americans and PAA could be substantially different than the conventional cut off limits defined by MetS using the NCEP-ATP III or IDF criteria as demonstrated by several studies in PAA [23,35]. Gaillard *et al.* [27], observed that, insulin resistance did not change with increasing serum triglyceride levels in African American women divided into tertiles of triglycerides. However, similar to other studies in black women from the U.S. [26,47,48], South African [34,39,40] and native Ghanaian women [4,24], serum triglycerides levels remain low despite the marked obesity as assessed by BMI (kg/m^2^) and waist circumference in these women. Thus, clearly, there is a dissociation between HDL and triglycerides in PAA. We have thus referred to this phenomenon as the Insulin Resistance-Lipid Paradox in People of African Ancestry [37].

### 2.5. Metabolic Syndrome, Obesity and Hypertension in Black PAA

Obesity is a well-recognized risk factor for hypertension. According to the NHANES 1999–2004, over half of the people with hypertension are obese [49,50]. Hypertension is more common in African Americans than white Americans and occurs in approximately 40%–45% *vs.* 20%–25%, respectively [49,50]. For example, Harris *et al.* [51] found that the prevalence of hypertension was twice as high among African Americans when compared to whites. Hypertension is considered a major cause of CVD morbidity and morbidity in African Americans.

The exact cause of hypertension in African Americans remains uncertain, but it is suspected to be multifactorial involving genetics and environment [49,50,51,52]. Recent studies have suggested insulin resistance as the pivotal lesion for hypertension in certain populations [49,50], but this remains controversial in African Americans. While hypertension is associated with insulin résistance in Caucasians, it has been difficult to establish such a strong association in African Americans and PAA in general [50,51,52,53]. While the prevalence of MetS was similar among African Americans and whites, Lteif *et al.* [25] reported higher rates of hypertension, insulin resistance, waist circumference and endothelial dysfunction in African Americans than in whites in their study. The author concluded that additional (but ill-defined) variables contributed to the endothelial dysfunction in African Americans and thus may contribute to the higher CVD morbidity and mortality. Therefore, based on existing evidence, we can categorically state that hypertension is extremely dangerous in PAA, especially young black males. In addition to its association with CVD, it is considered a major cause of chronic kidney disease resulting in end stage renal failure and heart failure [53,54,55]. Most importantly, the presence of glucose intolerance and diabetes increases the CVD outcomes in hypertensive African Americans [37,38,53,54]. Together with obesity and type 2 diabetes, hypertension contributes to the morbid CVD outcomes in African American women and the 2–4 fold higher CVD mortality and morbidity rates than white women [49]. Indeed, diabetes accounts for 50% of attributable causes of End Stage Renal Failure requiring dialysis in the USA [54,55]. Most importantly, approximately 45%–50% of these patients requiring dialysis are African Americans [54,55]. Furthermore, the major predisposing risk factors for hypertension are genetic and environmental. Unfortunately, the recent decline in CVD mortality and morbidity over the past three decades in the U.S. white population has not equally benefited African Americans, especially women, perhaps due in part to inability to achieve normal blood pressure in hypertensive African Americans [51,52,53,54,55]. However, the higher CVD mortality in hypertensive African Americans has been partly attributed to higher rates of obesity, type 2 diabetes, physical inactivity, socioeconomic factors and lack of access to appropriate health care, especially when compared with whites.

We have reported that the relation between insulin resistance and blood pressure is very weak in African Americans unlike white Americans [37,52]. Thus, we have termed the dissociation between insulin resistance and blood pressure as the Insulin Resistance and BP paradox in African Americans [37].

### 2.6. Insulin Resistance—Blood Pressure Paradox in African Americans

Several studies have reported that hypertension is also associated with greater waist circumference in PAA than whites [7,8,15,16,26,52,53]. In our studies, examining MetS and hypertension among African American women, we found that both systolic and diastolic blood pressure were associated with waist circumference, but not BMI and % body weight [51]. Thus, the conventional notion that insulin resistance is pivotal for CVD and hypertensive events does not appear to be supported in African American women, consistent with several previous reports in insulin resistant, nondiabetic, obese African Americans [20,21,25] and Black South African women [23,31,34,39,40], and native Ghanaians [4,24] in contrast with those of white women. High blood pressure or its surrogate e.g., chronic kidney disease, microalbuminuria are more prevalent in black PAA than whites. Both hypertension and chronic kidney disease, seem to carry remarkably strong negative impact on CVD outcomes in PAA. Thus, in the contest of black PAA, it appears that hypertension carries more burden as well as risk for CVD and other chronic complications. Thus, we can surmise that controlling hypertension in PAA, would be an important clinical outcome that would lower risk for CVD. Long-term studies of PAA across the African Diaspora are needed to confirm this hypothesis.

### 2.7. Metabolic Syndrome, Glucose Intolerance, Pre-Diabetes and Type 2 Diabetes in Black PAA

It has been proposed that the MetS is a powerful determinant or predictor of diabetes. Elevated fasting serum glucose levels and pre-diabetes (impaired fasting glucose and/or impaired glucose tolerance) are associated with greater CVD events and stroke as well as the associated morbidity and mortality than people without diabetes [38].

Previous epidemiological studies have shown an association between hemoglobin A1C (A1C) and CVD outcomes, but this has been inconsistent in clinical trials. The A1C has been accepted worldwide as an indicator of range of blood glucose over the preceding 2–3 months. A1C is considered normal (<5.6%), prediabetes (>5.7%–<6.4%) and diabetes (≥6.5%) according the American Diabetes Association. A1C reflects and predicts the Mets [56]. We [56] and others [57,58,59,60] have postulated that the A1C could serve as a surrogate of MetS. Osei *et al.* [56] studied healthy African Americans with family history of type 2 diabetes, divided according to tertiles of A1C. Subjects in the upper tertile had a mean A1C of 5.8%, that correlated with significantly higher fasting glucose (albeit within normal limits) and lower insulin sensitivity (Si and HOMA-IR) and significantly greater mean systolic and diastolic blood pressure than those in the lower tertiles. These were not explained by differences in body composition. The authors concluded that the higher A1C selected out individuals who were perhaps genetically unique to develop MetS as currently defined by NCEP-ATP III criteria. It is worthy to note that, African Americans have greater AlC at every level of glucose tolerance than whites [57,58,59,60]. Herman *et al.* [58] examining ethnic difference among subjects with IGT in the Diabetes Prevention Program, found A1C to be higher in African Americans (6.1%) compared to whites (5.9%). Alexander *et al.* [61] demonstrated in the NHANES study that, in adults >50 years of age, progressed from normal glucose tolerance, impaired glucose tolerance, impaired fasting glucose to diabetes, the corresponding prevalence of MetS also increased 28.5%, 33.1%, 71.3%, 86%, respectively. Similar results were found in African American subjects with IFG in the NHANES III [61] and in children in the NHANES [60]. In addition, in their study, MetS and its associated risk factors were more common in the highest A1C levels, similar to those of Osei *et al.* [56] in African Americans and Saaddine *et al.* [60] in children in the NHANES data. Data examining the relationship between prediabetes or its surrogates (IFG or IGT) and MetS are scant in studies in blacks in SSA. This is mainly due to lack of availability of A1C technology and differences in data collection methodologies. Amoah *et al.* [4] found that diabetes to be common among urban Ghanaian’s and that this also correlated with higher rates of obesity in their population. In addition, Oladapo *et al.* [28] in a population of Native Nigerians’ found higher rates of type 2 diabetes and corresponding MetS in their subjects. Kalk *et al*. [62] found prevalence of MetS to be 46.5%* vs.* 74.1% in type 2 diabetic, blacks and whites South Africans with type 2 diabetes. Data from the International Diabetes Federation Diabetes Atlas demonstrate the largest increase in diabetes and related diseases of glucose metabolism to occur in developing regions of the world including Africa. Thus, glucose intolerance and diabetes are major features of the MetS that should be evaluated and treated to prevent major CVD complications.

## 3. Metabolically Healthy Obese (MHO) and Metabolically UnHealthy Obese (MUHO) Black PAA

Recently there has been interest in obese individuals who manifest divergent metabolic consequences. Some obese subjects have metabolic characteristics consistent with the MetS whereas others do not. We [17,18,19] and others [20,21,25,46,47,48] have shown that PAA are more insulin resistant than whites. However, not all obese PAA are insulin resistant. Banergi *et al.* [63] found insulin resistant and insulin sensitive variants in African Americans with type 2 diabetes. No data were provided in the nondiabetic subjects in their study, nor are we aware of similar data in the literature. In other populations of African ancestry, Jennings *et al.* [34] found that central fat distribution, total adiposity, and physical activity distinguished between insulin sensitive and insulin resistant nondiabetic black South African American women. Gaillard *et al.* [64] found that 33% of nondiabetic healthy African American women were insulin sensitive and constituted MHO while 67% had insulin resistance and constituted the MUHO, based on our empirical definition of insulin resistance (Si) of ≥2.7 and <2.7 (×10^−4^ × min^−1^(μU/mL)^−1^), respectively. Thus, we showed clearly that significant proportion of obese African American women are insulin resistant. This is of interest since insulin resistance is regarded as the underpinning of CVD, hypertension, triglycerides and MetS in other populations [25,40,48,49,50,51]. Thus, theoretically, African American women should have generally higher prevalence of MetS than their white counterparts. This is however not the case.

The role of excessive adiposity in CVD risk is unclear. Recent studies have shown that obesity was not associated with an increased risk of future cardiovascular events among individuals without the MetS, but among individuals with the MetS, obesity was associated with an increase in CVD risk [21,25,32,33,34,35,36]. Wildman *et al.* [65] using the NHANES data found that prevalence of metabolic abnormalities were similar among normal weight metabolically health and obese metabolically unhealthy adults. In this study, age and waist circumference contributed to the MUHO subjects.

## 4. Implications of Obesity in MetS and Its Components in Black PAA

We have attempted to address the inconsistencies and disparities in MetS and each of the five components of the NCEP-ATP III criteria in black people of the African Diaspora. This issue is very important since the current NCEP-ATP III criteria assumes that all the five parameters are equally important in their ability to identify cardiovascular risk factors or predict CVD outcomes in black PAA. However, we [17,18,19,27,37,52] and others [20,21,23,24,25,26,30,31,32,33,34,35,36] have shown that in black PAA the contributions of each of the varying components vary by gender and ethnicity. We have proposed that each of the five components should be weighted differently in black PAA based upon their ability to predict risk for CVD. We have shown that each of the components of MetS could have differential impact on CVD morbidity and mortality in a given ethnic/racial population.

## 5. Conclusions

In summary, the causes as well as the mechanisms of the paradoxical relationships of the MetS are unknown. These risk factor appear to tract from childhood to adulthood and across the African Diaspora. Given these observations we attempted to conclude that the HDL/Triglyceride relationship is predominately inherited in black PAA. The MetS offers a tool that can be used by primary health care providers to identify black PAA who are at risk for CVD and type 2 diabetes. We have proposed that each of the components in the NECP-ATP III criteria and cut-off points and time-course should possibly be weighed differently in PAA because of the dissociation of CVD risk factors and the associated mortality and morbidity in black people of African ancestry [37]. We have further suggested that the prevalence (and perhaps the CVD impact) of the various components of MetS and MetS *per se* may not be uniform in all ethnic/racial groups. Indeed, the components of MetS, such as hypertension and obesity which are more common in black PAA, appear to carry much greater CVD morbidity and mortality, especially among women. Therefore, further studies in black PAA, especially those living in Sub-Saharan Africa, are needed to establish the correct criteria and individual components for MetS to identify high risk individuals of African ancestry.
